# Metabolic and Physiological Predictors of Enteric Methane Emissions in Early Lactation Dairy Cows: A Prospective Observational Study

**DOI:** 10.3390/life15121815

**Published:** 2025-11-27

**Authors:** Justina Krištolaitytė, Karina Džermeikaitė, Lina Anskienė, Samanta Grigė, Akvilė Girdauskaitė, Arūnas Rutkauskas, Ramūnas Antanaitis

**Affiliations:** 1 Large Animal Clinic, Veterinary Academy, Lithuanian University of Health Sciences, Tilžės Str. 18, LT-47181 Kaunas, Lithuania; karina.dzermeikaite@lsmu.lt (K.D.); samanta.grige1@lsmu.lt (S.G.); akvile.girdauskaite@lsmu.lt (A.G.); arunas.rutkauskas@lsmu.lt (A.R.); ramunas.antanaitis@lsmu.lt (R.A.); 2 Department of Animal Breeding, Veterinary Academy, Lithuanian University of Health Sciences, Tilžės Str. 18, LT-47181 Kaunas, Lithuania; lina.anskiene@lsmu.lt

**Keywords:** methane emission, innovative technologies, dairy cows, blood samples

## Abstract

This study aimed to investigate the relationship between enteric methane (CH_4_) emissions and metabolic, physiological, and behavioural factors in early lactation Holstein cows. Forty-two cows were observed over a span of five consecutive weeks (0–100 days in lactation). CH_4_ concentration (ppm) was quantified with a portable laser detector, whereas rumination duration, temperature, and water consumption were documented using intraruminal boluses. Weekly blood samples were examined for beta-hydroxybutyrate (BHB), C-reactive protein (CRP), urea (UREA), lactate dehydrogenase (LDH), aspartate aminotransferase (AST), and gamma-glutamyl transferase (GGT) levels. The evaluation of milk yield and composition was conducted utilising in-line infrared sensors. Cows were classified against clinical reference intervals, and associations were tested via group comparisons, correlations, multiple linear regression, linear mixed models (cow ID random effect), ROC analysis, and by relating CH_4_ to dry matter intake (DMI). Cows with elevated BHB (≥1.2 mmol/L) emitted 87.8% more CH_4_ than cows within range and showed higher CH_4_ yield per kg DMI; elevated GGT was likewise associated with higher CH_4_ (+25.2%). CH_4_ correlated positively with BHB (r = 0.54, *p* < 0.01), and negatively with rumination (r = −0.38, *p* < 0.05). Regression explained 30.2% of CH_4_ variance (adjusted R^2^ = 0.302): BHB was a positive predictor (β = 0.55, *p* = 0.047), whereas LDH was negative (β = −0.21, *p* = 0.033). A three-way interaction (BHB group × AST × GGT) was significant in the mixed model (F = 6.91, *p* = 0.002). For discrimination of high emitters, BHB achieved AUC = 0.889; among on-farm traits, milk yield (AUC = 0.823) and lactose (AUC = 0.701) performed best. DMI related inversely to CH_4_ yield (r = −0.69, *p* = 0.058). The findings indicate that enteric methane production during early lactation is not exclusively influenced by diet but is significantly associated with systemic metabolic health. Integrating physiological and production characteristics may improve precision-driven methane monitoring and mitigation strategies in dairy systems.

## 1. Introduction

Greenhouse gas (GHG) emissions from dairy farming have been acknowledged as a significant factor in climate change, especially considering policy measures like the European Green Deal, which prioritises the reduction in agricultural methane (CH_4_) emissions as a crucial approach to attain climate neutrality by 2050 [1]. Ruminant livestock, particularly cattle and sheep, account for up to 18% of global greenhouse gas emissions, predominantly due to enteric CH_4_ production [2]. Ruminant livestock, particularly cattle and sheep, account for up to 18% of global greenhouse gas emissions, predominantly due to enteric CH_4_ production. A single mature dairy cow can produce up to 500 litres of CH_4_ daily, and CH_4_ has a global warming potential roughly 25 times that of carbon dioxide (CO_2_) [3,4]. Ruminants are biologically equipped to transform fibrous plant matter into high-quality animal protein; however, this process inevitably produces significant CH_4_ as a by-product of rumen fermentation [5].

Notwithstanding progress in measurement technologies, significant knowledge gaps remain concerning the relationships between enteric CH_4_ emissions and alterations in physiological, behavioural, and haematological parameters—especially during the transition period, characterised by marked metabolic and endocrine adaptations [6]. Comprehending the correlation between CH_4_ production and the metabolic health and adaptation mechanisms of cows during early lactation is thus of significant scientific and practical importance.

Early lactation is a crucial period in dairy cow physiology, characterised by a discrepancy between energy intake and expenditure that frequently results in negative energy balance (NEB), intensified by diminished dry matter intake [7,8]. Rumination and effective digestion are crucial for maintaining energy levels during this time. Since CH_4_ emissions represent an energetic loss during digestion, increased CH_4_ production may diminish net energy availability, potentially undermining immune function and heightening vulnerability to metabolic and infectious diseases [9,10].

Liver function plays a central role in these adaptations. It governs gluconeogenesis, protein and fat metabolism, and detoxification—processes that directly influence digestive efficiency, immune status, and ruminal fermentation. Biochemical blood indicators, such as the activity of liver enzymes including aspartate aminotransferase (AST), gamma-glutamyl transferase (GGT), and lactate dehydrogenase (LDH) are widely used to assess metabolic balance in dairy cattle [11,12]. These enzymes are among the most sensitive biomarkers reflecting liver function—AST primarily indicating hepatocellular necrosis, while GGT reflects cholestasis—and are therefore considered standard parameters for detecting and diagnosing hepatic injury or dysfunction [13]. LDH, although non-organ-specific [13,14,15]. Thus, monitoring these enzymes provides a valuable tool for evaluating subclinical liver dysfunction, which could influence rumen metabolism and CH_4_ output.

Alongside standard liver enzymes, C-reactive protein (CRP)—a major acute-phase protein (APP)—has gained attention as a sensitive indicator of systemic inflammation and metabolic stress in dairy cows. Synthesised by the liver in response to cytokines such as IL-1, IL-6, and TNF-α, CRP levels rise rapidly under physiological stress, including parturition, systemic inflammation, or metabolic disorders [16,17]. Compared to classical haematological markers, CRP may offer greater diagnostic sensitivity for early detection of metabolic imbalance in cows [18]. Although CRP has been widely studied in conditions like mastitis, recent findings highlight its relevance for assessing broader metabolic disturbances in transition cows, especially when interpreted alongside other metabolic and milk-based indicators [17,19,20,21].

The connection between energy balance in dairy cows and metabolic blood markers (including BHB, AST, GGT) and milk composition has been demonstrated [17]. A comprehensive observational study of high-yielding Holstein cows validated these associations, revealing that increased levels of metabolic and inflammatory biomarkers—such as BHB, AST, GGT, and acute-phase proteins—correlated with diminished milk yield, lactose content, and milk urea concentrations, illustrating the impact of subclinical inflammation and metabolic stress on lactational performance [22].

These findings underscore the need for accessible, non-invasive, and automated tools for on-farm monitoring of cow health and metabolic status. One promising development is the use of milk as a diagnostic biofluid—readily collected during routine milking and reflective of metabolic shifts via changes in composition [23]. Indicators such as the fat-to-protein ratio, lactose concentration, and somatic cell count have been widely recognised as useful proxies of energy balance and health [24].

In parallel, continuous behavioural data—including rumination duration and activity levels—may also reflect early signs of metabolic stress or discomfort [25]. Bolus technologies now enable continuous, in-line monitoring of internal physiological parameters, such as reticulorumen temperature, rumination, physical activity and drinking behaviour [26]. These tools, when combined with milk and blood monitoring, support early detection of metabolic disturbances and more targeted herd-level interventions [27].

Importantly, these physiological and behavioural indicators are also closely linked to feed intake efficiency—a key determinant of enteric CH_4_ production. Reduced rumination and lower activity may reflect compromised feed utilisation, potentially increasing CH_4_ yield per unit of dry matter intake (DMI). As highlighted by Zetouni et al. [28], feed digestibility and DMI are key modulators of CH_4_ emissions, with more efficient cows producing less CH_4_ per kg of milk [29,30]. Therefore, the integration of behavioural monitoring with milk composition and CH_4_ data can offer insights not only into health status but also into emission efficiency, supporting dual goals of improved animal welfare and climate mitigation.

This study intended to examine the correlation between the metabolic condition of early-lactation dairy cows and their enteric methane emissions. Repeated measurements of CH_4_ output, blood biomarkers (including BHB, AST, GGT, LDH, CRP, physiological indicators, and milk composition were analysed to determine whether deviations from established physiological reference ranges are associated with altered CH_4_ production. The study also explored the potential of routinely monitored health indicators as predictive markers of CH_4_ emission intensity in dairy cows.

## 2. Materials and Methods

### 2.1. Housing Conditions of Study Animals

This study was conducted in full compliance with the Lithuanian Law on Animal Welfare and Protection and obtained ethical approval under ethical permit NO. PK012858 from the Department of Anatomy and Physiology of LSMU (Approval date: 6 June 2017). The research was carried out on a commercial dairy farm in Lithuania (latitude 54.906851°, longitude 23.961297°), housing around 500 lactating cows in free-stall barns. The study was conducted from 16 September to 30 October 2024 and involved 42 clinically healthy multiparous Holstein cows. The animals exhibited an average parity of 2.7 ± 0.6 and a mean body weight of 650 ± 45 kg (SE). The selection criteria were determined by clinical health, production records, and the absence of clinical or subclinical metabolic disorders at the trial’s commencement. The experimental period encompassed the early lactation phase, commencing immediately post-calving and extending until 100 days in milk (DIM).

Cows were housed in a loose-housing system and allocated into two management groups within mechanically ventilated free-stall barns (DeLaval Inc., Tumba, Sweden) to guarantee suitable microclimatic conditions. Cows were milked voluntarily using an automated milking system, with unrestricted access to the milking robots (DeLaval Inc., Tumba, Sweden) at any time. On average, cows entered the milking system approximately twice per day.

During the study, cows were administered a nutritionally balanced total mixed ration (TMR) designed to fulfil the nutrient needs of early-lactation dairy cows. The ration was provided bi-daily at 08:00 and 16:00, accompanied by unrestricted access to potable water. The dietary cation–anion difference (DCAD) of the total mixed ration (TMR) was approximately +250 mEq/kg dry matter to facilitate rumen function and maintain milk production during the postpartum phase. The comprehensive ingredient composition and chemical profile of the TMR are delineated in Table 1 and Table 2. In their prior lactation, the enrolled cows yielded an average of 10,304 kg of energy-corrected milk, comprising 4.1% fat and 3.4% protein, as determined by standard reference equations.

### 2.2. Experimental Animals and Study Design

42 clinically healthy multiparous Holstein cows were chosen for the study immediately following calving. All cows calved without complications during the preceding lactation and exhibited an average parity of 2.8 ± 0.6. A licensed veterinarian conducted daily observations of the animals to verify the absence of clinical disorders throughout the study period. Observations were performed from calving until 100 days in milk (DIM), denoting the early lactation phase, a time marked by significant metabolic and physiological adaptations. The research employed a repeated-measures observational design, wherein each cow acted as its own control. No experimental treatments or interventions were administered. This design facilitated the assessment of intra-animal variations over time, minimising inter-subject variability and augmenting the dependability of correlations between physiological and behavioural parameters.

Two precision livestock technologies were employed to acquire extensive data on production, metabolic, and behavioural indicators. The BROLIS HerdLine in-line milk analyser (Brolis Sensor Technology, Vilnius, Lithuania) continuously assesses milk composition characteristics, including concentrations of fat, protein, and lactose, in addition to measuring milk temperature and yield. Simultaneously, SmaXtec boluses (SmaXtec Animal Care GmbH, Graz, Austria) were used to evaluate internal body temperature (°C), rumination duration (min/day), water consumption (L/day), reticulorumen pH, and activity levels (h/day). Furthermore, blood samples were obtained to evaluate biochemical parameters indicative of metabolic and hepatic function status.

The study offered a comprehensive perspective on the correlation between dairy cow health and the emission of enteric CH_4_ by integrating milk composition, physiological, and behavioural data. The repeated-measures design permitted each cow to function as its own control, thereby strengthening the observed associations and facilitating within-animal comparisons over time.

### 2.3. Measurement of Variables

#### 2.3.1. Methane Emission Measurement

Enteric methane emissions were assessed using a portable Laser Methane Smart detector (GASTAR Co., Ltd., Yamato, Kanagawa, Japan). The instrument operates on the principle of infrared absorption spectroscopy, enabling real-time quantification of CH_4_ by analysing laser light reflected from the target surface. The obtained readings express CH_4_ concentration as column density (ppm × m), which represents the product of the average CH_4_ concentration (ppm) and the laser beam path length (m). The Laser Methane Smart is a non-invasive, portable instrument designed to detect CH_4_ concentrations from 1 to 50,000 ppm × m, with an accuracy of ±10% within the 100–1000 ppm × m range, a response time of 0.1 s, and an effective detection range of 0.5–30 m [31,32]. The device autonomously calibrates with an internal reference cell containing CH_4_ and conducts self-assessments during both initialisation and operation, thus obviating the necessity for external calibration processes [31]. Enteric CH_4_ was measured using a handheld laser methane detector while cows were resting in lying stalls. The operator accessed all cows from the same fixed standing position at the front of the stalls, ensuring a consistent distance of approximately 1 m from the nostrils. The laser beam was manually aligned to the nostril area to capture exhaled air containing gases released during respiration and eructation [33]. All measurements were performed by a single trained veterinarian to minimise variability caused by handling. Each measurement session endured a minimum of five minutes to capture the full eructation cycle, as recommended by previous studies [34]—during which approximately 360–720 data points were recorded per cow at a sampling frequency of 0.5–1.0 s [35]. Measurements were conducted twice daily, and this schedule was maintained consistently across the five-week study period to capture potential periodic fluctuations and ensure representative data. This protocol facilitated the collection of one to two CH_4_ measurements per second. All measurements were performed under low-wind conditions and at a uniform time—approximately two hours after the morning feeding—to mitigate diurnal variations in CH_4_ emissions [36]. The angle and distance between the detector and the cow’s nostrils were standardised across all sessions, and reflective surfaces were eliminated to reduce environmental interference. This method is consistent with established techniques for laser-based CH_4_ detection, which have shown a significant correlation with reference methods like respiration chambers (R^2^ ≈ 0.9), thereby affirming the precision and dependability of laser CH_4_ detectors for real-time evaluation of enteric emissions on farms without interfering with typical animal behaviour [32].

#### 2.3.2. Blood Sample Analysis

Blood samples were collected once per week from each cow, approximately four hours after the morning milking and feeding. Sampling coincided with routine clinical evaluation, during which cows were restrained in a resting stall or headlock to allow safe venipuncture. A small volume of blood was drawn from the coccygeal vein using a sterile needle and syringe and transferred into 10-mL vacuum tubes without anticoagulant (BD Vacutainer^®^, Eysin, Switzerland) for subsequent biochemical analysis. The samples were immediately transported to the Laboratory of Clinical Tests at the Large Animal Clinic, Veterinary Academy, Lithuanian University of Health Sciences, maintained at +4 °C, and processed within one hour of collection. In the laboratory, serum was obtained by centrifugation for 15 min at 1500× *g*. The concentrations of β-hydroxybutyrate (BHB), C-reactive protein (CRP), urea (UREA), lactate dehydrogenase (LDH), aspartate aminotransferase (AST), and gamma-glutamyl transferase (GGT) were determined using a Hitachi 705 automated analyser (Hitachi, Tokyo, Japan) with DiaSys reagents (Diagnostic Systems GmbH, Berlin, Germany).

#### 2.3.3. Cow Behaviour

For real-time physiological monitoring, each of the 42 study cows was orally administered a SmaXtec bolus (SmaXtec Animal Care Technology^®^, Graz, Austria) within the initial 30 days postpartum. The boluses were activated before insertion, associated with the cow’s unique ear tag, and linked to the central base station system. The administration was conducted by the same experienced veterinarian using a special applicator, in accordance with the manufacturer’s guidelines. The gravity-based boluses were engineered to remain in the reticulum for the entirety of the study.

These sensor-integrated boluses continuously monitored reticulorumen temperature (RT), rumination duration (min/day), physical activity (h/day), and water consumption. Data were gathered at 10 min intervals and wirelessly transmitted to the SmaXtec Messenger^®^ software (version 4) through antenna receivers installed in the barn. The microprocessor-controlled system utilised an analogue-to-digital (A/D) converter to digitise data, which was subsequently stored on an external memory chip for further analysis.

Water consumption was assessed using proprietary AI algorithms that analyse temperature reductions in the reticulum after drinking events, facilitating indirect, non-invasive measurement of hydration patterns. This approach obviates the necessity for supplementary monitoring infrastructure while providing individual-level insights into drinking behaviour.

During the trial, these boluses delivered continuous, real-time data on each cow’s physiological and behavioural condition, facilitating early identification of potential health concerns and improving the accuracy of animal management.

#### 2.3.4. Milk Parameters

The composition of milk was consistently monitored using the BROLIS HerdLine in-line analyser (Brolis Sensor Technology, Vilnius, Lithuania), which was directly integrated into the milking system. This device utilises a GaSb broadly tuneable external cavity laser spectrometer functioning within the 2100–2400 nm spectral range. Operating in transmission mode during milk flow, it facilitates real-time, individual-level analysis of each milk sample collected during milking. The system functions as a compact, on-farm spectroscopic laboratory by capturing molecular absorption spectra, enabling precise quantification of essential milk components, including fat, protein, and fat-to-protein ratio (F:P).

The accuracy of the BROLIS HerdLine analyser was independently verified and calibrated at the Eurofins laboratory. The calculated root mean square error of prediction (RMSEP) values were 0.21% for milk fat, 0.19% for protein, and 0.19% for lactose, confirming a high analytical precision of the instrument.

#### 2.3.5. Individual Dry Matter Intake (DMI) Calculation

Daily evaluations of feed consumption were conducted to assess the correlation between individual dry matter intake (DMI) and CH_4_ emissions. Feed intake for each cow was assessed by measuring the total feed provided before feeding and the leftover feed after 24 h. The actual daily feed intake was calculated by subtracting the refusals from the quantity offered. The individual DMI values were subsequently calculated using the following equation:

$${DMI}_{i}=\frac{{Feed\;Intake}_{i}\times DM\;\%\;of\;Diet}{100}\;$$

where DMI*_i_* is the estimated daily dry matter intake of individual cow (kg), Feed Intake*_i_* is the total feed consumed per cow (kg/day), and DM % of Diet is the dry matter percentage of the total mixed ration (TMR).

### 2.4. Duration of Experimental Observation

During the early lactation period, data were systematically collected from calving until 100 days in milk (DIM) and later analysed retrospectively. Each cow served as its own control within a repeated-measures observational design. Cows were retrospectively assigned to two groups based on whether their blood biomarker values were within or above the established reference intervals. Reference intervals for biochemical parameters vary across published sources and depend on analytical methodology and manufacturer characteristics. In this study, reference intervals correspond to ranges routinely used in our diagnostic laboratory for the Hitachi 705 analyser with DiaSys reagents, and they are consistent with values reported in the literature. For analysis purposes, values exceeding the upper limit of the reference interval were interpreted as elevated.

This post hoc grouping was performed after all laboratory analyses were completed, as deviations from physiological norms could only be identified at that stage. This approach enabled the evaluation of potential associations between metabolic alterations and enteric CH_4_ emissions. Therefore, the findings should be interpreted as exploratory. Blood parameters were specifically compared to established reference intervals for clinically healthy dairy cows (Table 3).

Cows that displayed at least one parameter exceeding the reference interval at any sampling point were categorised as “above normal,” whereas cows with values consistently within physiological limits were designated as “within normal range.” This stratification facilitated the identification of potential correlations between physiological status and CH_4_ emissions in the absence of visible clinical disease.

### 2.5. Statistical Analysis

All statistical analyses were conducted using IBM SPSS Statistics for Windows, version 29.0 (IBM Corp., Armonk, NY, USA). The Shapiro–Wilk test was applied to evaluate the normality of continuous variables. Data are expressed as means ± standard error of the mean (SEM), unless stated otherwise.

Multiple statistical methods were used to assess the correlation between CH_4_ emissions and specific blood, behavioural, and milk-related parameters. Pearson’s correlation coefficients were computed to assess associations among variables. To account for multiple comparisons, *p*-values were adjusted using the FDR method.

A multiple linear regression analysis was conducted to identify significant predictors of CH_4_ concentration among biochemical parameters, and collinearity diagnostics were performed to verify the model’s stability.

Linear mixed-effects models (LMMs) were developed to accommodate repeated measurements from individual cows and to evaluate the fixed effects of blood parameter groupings and their interactions on CH_4_ emissions.

The mixed model used to analyse CH_4_ emissions can be represented as:

Yijk = μ + αi + βj + (αβ)ij + γk + εijk

where Yijk is the methane emission for subject k under group i and condition j, μ is the overall mean, αi is the fixed effect of BHB groups, βj is the fixed effect of factor (UREA, AST, GGT, CRP, LDH, Rumination), (αβ)ij is the interaction effect between BHB groups and the factor, γk is the random effect for subject kk (cow_ID), and εijk is the residual error.

The model includes repeated measures across trials with an unstructured covariance matrix to account for within-subject variability. Linear mixed models were estimated using the default REML method in SPSS.

The Receiver Operating Characteristic (ROC) curve analysis was employed to assess the diagnostic efficacy of specific blood, behavioural, and milk traits in differentiating between cows with elevated and diminished CH_4_ emissions. The area under the curve (AUC) was classified as follows: 0.5–0.7 = poor, 0.7–0.8 = fair, 0.8–0.9 = good, and >0.9 = excellent discrimination.

A *p*-value below 0.05 was considered statistically significant for all analyses (*p* < 0.05).

## 3. Results

### 3.1. Group Comparison by Blood Biomarkers in Dairy Cattle

Dairy cows were categorised into two groups based on whether their blood biomarker concentrations fell within or outside the reference intervals (Table 3). Group 1 included cows with biomarker values within physiological norms, while Group 2 comprised cows with elevated values (above or below the reference threshold, depending on the marker). This binary classification was applied separately for each biomarker (e.g., BHB < 1.2 mmol/L = Group 1; BHB ≥ 1.2 mmol/L = Group 2).

#### 3.1.1. Comparison of Traits Between BHB Groups

Cows were divided into two groups based on their BHB activity: Group 1 consisted of cows with BHB levels within the reference range (<1.2 mmol/L), while Group 2 included cows with elevated BHB values (≥1.2 mmol/L). Descriptive statistics for each group are presented in Table 4.

Dairy cows were grouped according to blood β-hydroxybutyrate (BHB) concentration, using a threshold of 1.2 mmol/L to distinguish cows with normal (Group 1) and elevated (Group 2) levels. Group comparisons revealed several statistically significant differences in physiological, metabolic, and productive traits (Table 4).

CH_4_ emission reveals a notable difference between the two groups, where CH_4_ is significantly 87.8% higher in the group of cows with elevated BHB (≥1.2 mmol/L) (Group 2) compared to low BHB cows (Group 1). The comparative analysis of BHB demonstrated that BHB concentration itself differed markedly between the groups, with Group 2 cows exhibiting a 230% higher mean BHB value (*p* < 0.001).

Urea values revealed a statistically significant difference where Group 2 exhibited 8.74% higher mean value compared to Group 1 (*p* < 0.05). Although mean AST, GGT, CRP, and LDH values were numerically higher in Group 2, only LDH approached a moderate effect size (ω^2^ = 0.025), suggesting some hepatic involvement in cows with elevated BHB.

Among the traits analysed, milk temperature showed the largest effect size (ω^2^ = 0.128), suggesting a strong association with BHB group classification, although mean values remained within normal physiological ranges.

Milk protein content (ω^2^ = 0.052) and milk lactose (ω^2^ = 0.019) were also slightly lower in cows with elevated BHB, which may reflect the impact of metabolic imbalance on milk synthesis. In contrast, traits such as milk yield, rumination time, and water intake exhibited negligible or negative omega-squared values, implying minimal differentiation across BHB groups (Table 4).

#### 3.1.2. Comparison of Traits Between AST Groups

Cows were divided into two groups based on their serum aspartate aminotransferase activity: Group 1 consisted of cows with AST levels within the reference range (<125 U/L), while Group 2 included cows with elevated AST values (≥125 U/L). Descriptive statistics for each group are presented in Table 5.

The statistical analysis of the traits investigated demonstrated a significant variation in mean values, indicating a robust differentiation across the examined AST groups. The average GGT was 17.66% higher in cows with elevated AST (Group 2) compared to those in the normal range (Group 1) (*p* < 0.001). Similarly, the average of LDH was 22.69% higher in Group 2 (*p* < 0.001), indicating a close association between AST and other liver function enzymes. CRP average value was significantly elevated in Group 2, with an 11.0% increase compared to Group 1 (*p* < 0.05). Additionally, the milk temperature average value was 0.8% higher in the second group of AST, compared to the first (*p* < 0.05).

High effect values for GGT (ω^2^ = 0.121) and LDH (ω^2^ = 0.162) indicate moderate to large effect sizes, suggesting moderate to large associations between AST elevation and these liver-related biomarkers. CRP (ω^2^ = 0.029) and milk temperature (ω^2^ = 0.032) demonstrated small but notable effect sizes.

Other traits, including CH_4_ emission, rumination, activity, milk yield, milk composition, and reticuloruminal temperature, showed negligible or negative omega-squared values, suggesting limited or no differentiation between AST groups for these parameters (Table 5).

#### 3.1.3. Comparison of Traits Between GGT Groups

Cows were classified into two groups based on their serum gamma-glutamyl transferase activity, using a threshold of 50 U/L to distinguish normal (Group 1) from elevated (Group 2) values. Group-wise comparison revealed statistically significant differences in selected physiological and biochemical traits (Table 6).

CH_4_ emission was significantly higher in cows with elevated GGT (Group 2), with a 25.18% increase compared to those in Group 1 (*p* < 0.05). Additionally, AST and LDH—both liver-associated enzymes—showed marked increases in Group 2. Specifically, AST values were 27.0% higher (*p* < 0.01), and LDH levels were 22.1% higher (*p* < 0.001) compared to cows with normal GGT activity.

Some traits exhibited relatively high omega-squared (ω^2^) values, indicating moderate to large effect sizes and a greater proportion of variance explained by the factor. LDH (ω^2^ = 0.167), demonstrated a strong association with GGT groupings and AST (ω^2^ = 0.149) hepatic enzyme, also showed substantial variance explained by GGT levels. Traits such as CH_4_ (ω^2^ = 0.034), UREA (ω^2^ = 0.021), and water intake (ω^2^ = 0.007) showed small but positive effect sizes, indicating potential but limited sensitivity to GGT groupings.

CRP, rumination, rumen temperature, milk protein, milk lactose, milk temperature, activity, and BHB all had ω^2^ values below zero. These results suggest that these traits are either biologically independent of GGT-related processes or that the current sample lacked sufficient power to detect subtle group-level differences (Table 6).

#### 3.1.4. Comparison of Traits Between LDH Groups

Cows were classified into two groups based on their serum lactate dehydrogenase activity, using a threshold of 1500 U/L to distinguish normal (Group 1) from elevated (Group 2) values. Descriptive statistics for each group are presented in Table 7.

The statistical evaluation of the examined traits revealed a significant difference in mean values among the analysed LDH groups. The highest statistically significant average difference was detected in AST level; the mean AST level was 24.9% higher in Group 2 compared to Group 1 (*p* < 0.001), while the GGT concentration was 23.0% higher in the elevated LDH group (*p* < 0.01). Blood BHB concentration was also significantly higher in Group 2, with a 37.3% increase compared to Group 1 (*p* < 0.05). In addition, cows with elevated LDH showed a marginal but statistically significant increase in milk temperature (+0.8%, *p* < 0.05).

Two traits exhibited Omega-squared values exceeding the 0.05 threshold, with moderate effect sizes. AST (ω^2^ = 0.135) and GGT (ω^2^ = 0.112) showed the strongest association with LDH groupings, suggesting a shared physiological or pathological pathway or tissue damage. BHB (ω^2^ = 0.029) and milk temperature (ω^2^ = 0.034) showed small effect sizes but may still reflect metabolic disturbance.

Other traits, including CH_4_ emission, milk yield, milk protein, milk lactose, activity, rumination, and water intake, demonstrated negligible or negative ω^2^ values. These results indicate low sensitivity to LDH group classification or potential biological independence from LDH-associated processes.

Group-wise comparisons were not performed for CRP and UREA due to insufficient variation across individuals relative to the reference thresholds, which limited meaningful group stratification.

### 3.2. Correlation Analysis

**Figure 1:**
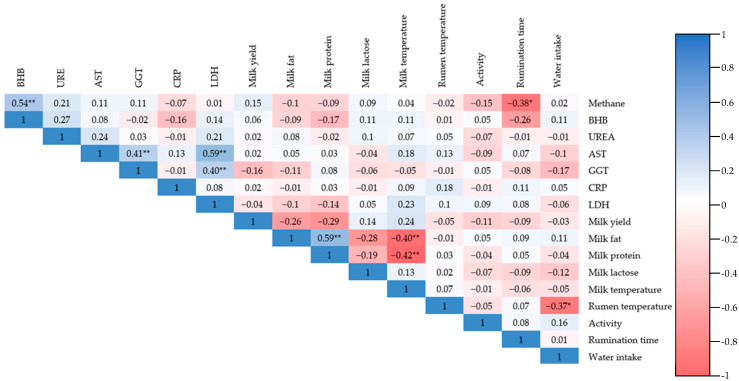
Correlation heatmap between investigated physiological, productive, and behavioural traits with statistical significance markers. *—Correlation r (Pearson’s correlation coefficient) is significant at the *p* < 0.05 level (2-tailed). **—Correlation r (Pearson’s correlation coefficient) is significant at the *p* < 0.01 level (2-tailed).

The correlation analysis revealed a range of significant associations between physiological, biochemical, productive, and behavioural traits in early-lactation dairy cows (Table 8). Most correlations were low to moderate in strength, yet several biologically relevant patterns emerged (Figure 1).

A moderate positive correlation was estimated between BHB and CH_4_ emissions (r = 0.54, *p* < 0.01), suggesting a potential link between energy metabolism and enteric CH_4_ fermentation processes.

A low negative significant relationship was detected between CH_4_ emissions and rumination (r = −0.38, *p* < 0.05), suggesting that increased rumination may be associated with reduced enteric CH_4_ output, potentially due to improved digestive efficiency or altered fermentation dynamics.

Among liver function indicators, AST was highly correlated with LDH (r = 0.59, *p* < 0.01) and GGT (r = 0.41, *p* < 0.01), indicating that liver function markers are closely interrelated and may jointly reflect systemic stress or inflammation.

The positive correlation between fat and protein content (r = 0.59, *p* < 0.01) reflects common nutritional and physiological drivers, such as energy intake and mammary gland function. This relationship is consistent with the co-regulation of milk solids under similar metabolic conditions.

Milk fat and milk temperature (r = −0.40, *p* < 0.05) and milk protein and milk temperature (r = −0.42, *p* < 0.05) suggest that higher milk solids are associated with lower milk temperature, potentially due to differences in secretion dynamics or thermal properties of milk components.

Higher rumen temperature is linked to decreased rumination (r = −0.37, *p* < 0.05), which may reflect changes in fermentation heat production or discomfort affecting chewing activity (Figure 1).

### 3.3. Multiple Linear Regression Analysis

A multiple linear regression model was used to predict CH_4_ concentration based on selected blood and metabolic biomarkers, demonstrating a moderate predictive capacity, with an R^2^ of 0.335 and an adjusted R^2^ of 0.302. This indicates that approximately 30.2% of the variance in CH_4_ levels can be explained by the included predictors: BHB, UREA, AST, GGT, CRP and LDH. Additionally, collinearity diagnostics confirmed the absence of problematic multicollinearity among predictors. The condition index remained below the critical threshold of 30 across all dimensions, and variance proportions did not show clustering of high values (*p* > 0.5) across multiple predictors in the same dimension. These diagnostics validate the stability and interpretability of the regression coefficients, reinforcing the reliability of the model’s findings.

Among the included variables, BHB emerged as a significant positive predictor of CH_4_ levels (β = 0.55, *p* = 0.047), suggesting that elevated ketone body concentrations may be associated with increased ruminal fermentation activity or altered energy metabolism that promotes CH_4_ production. Additionally, LDH (β = −0.21, *p* = 0.033) emerged as a significant negative predictor, suggesting that higher levels of this cellular damage marker are associated with lower CH_4_ output, possibly due to compromised metabolic efficiency or altered microbial function in the rumen under systemic stress. CRP (β = 0.02, *p* = 0.788) did not reach statistical significance, contrary to earlier interpretation (your previous version had it as a significant negative predictor). This appears to be a typo or mix-up. Based on the table, CRP is not a significant predictor in this model.

UREA (*p* = 0.341), AST (*p* = 0.286), and GGT (*p* = 0.071) also did not reach statistical significance, though GGT approached the threshold (*p* = 0.071), suggesting a potential trend worth further investigation in larger samples (Table 8).

### 3.4. Linear Mixed Model with Interaction Effects

To investigate more complex relationships between metabolic status and CH_4_ emissions, a linear mixed model was constructed. The model included interaction terms among blood biomarkers, with cow ID as a random effect to account for repeated measures. Interaction terms between markers were included. While most interactions were not significant, the mixed model analysis revealed a statistically significant intercept (F = 6.42, *p* = 0.015), indicating a strong baseline level of CH_4_ emissions across subjects. Among the interaction terms, the BHB group × AST × GGT interaction was highly significant (F = 6.91, *p* = 0.002), suggesting a synergistic effect between liver enzymes AST and GGT in modulating CH_4_ production within BHB groups.

Other biochemical and behavioural interactions, including UREA, Rumination, CRP, and LDH, did not reach statistical significance individually or in combination, *p* > 0.05, indicating a combined effect of liver enzyme activity and ketone body levels on CH_4_ emission (Table 9).

### 3.5. ROC Curve Analysis for Methane Prediction

To evaluate the diagnostic performance of physiological, biochemical and milk parameters in identifying high CH_4_ emitters, Receiver Operating Characteristic (ROC) analysis was conducted.

Among blood traits, BHB demonstrated excellent discriminatory ability (AUC = 0.889), suggesting it is a strong candidate biomarker for identifying cows with high CH_4_ emissions. This finding may reflect alterations in energy metabolism and ruminal fermentation associated with elevated ketone levels.

**Figure 2:**
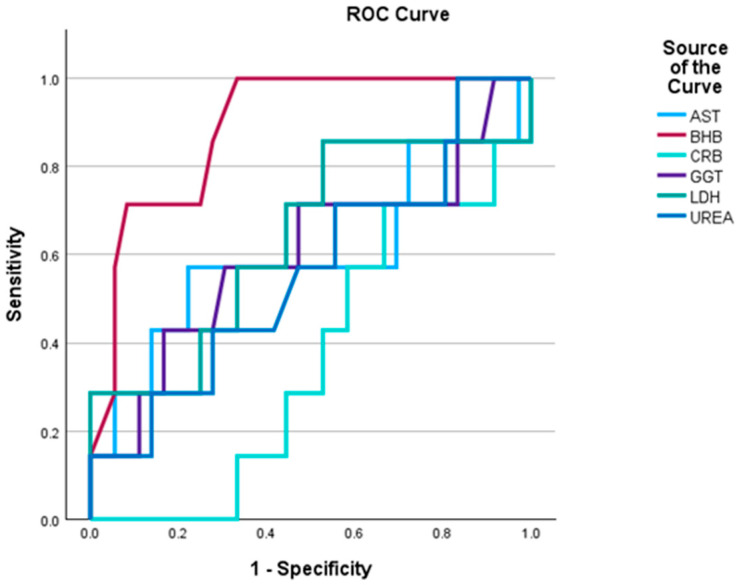
Comparative ROC curve analysis of blood traits for discriminating high methane emission.

GGT, AST, and UREA showed poor to fair discrimination (AUC range: 0.56–0.60), indicating limited diagnostic value when used in isolation, though they may contribute to multivariate models. In contrast, CRP and LDH yielded AUCs below 0.5, suggesting an inverse or non-informative relationship with CH_4_ emission. These markers may not be suitable for CH_4_ prediction in isolation (Figure 2).

**Figure 3:**
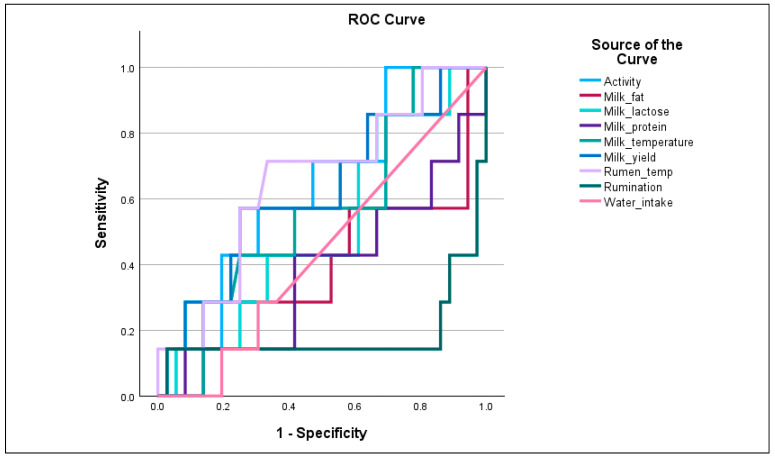
ROC curves for milk composition, rumen parameters, activity traits for discriminating high methane emission.

Among production and physiological parameters, milk yield (AUC = 0.823) emerged as the most effective trait for discriminating cows with high CH_4_ emissions, suggesting a strong association between productivity and CH_4_ output. This may reflect increased feed intake and rumen fermentation activity in high-yielding cows, contributing to elevated CH_4_ production. Milk lactose (AUC = 0.701) showed acceptable discriminatory ability, potentially associated with metabolic efficiency or rumen performance. Milk temperature, activity, and rumen temperature yielded an AUC near 0.5, indicating limited diagnostic value when used individually. Rumination (AUC = 0.183) and milk fat/protein (AUC < 0.4) demonstrated very poor or inverse discrimination, suggesting these traits may not be reliable indicators of CH_4_ emission status in isolation (Figure 3).

### 3.6. Influence of Dry Matter Consumption on Methane Emissions in Dairy Cows

While analysing behavioural patterns and their correlation with CH_4_ emissions, individual dry matter intake was evaluated to determine variations during the early lactation period and its possible effect on CH_4_ yield.

To further investigate the relationship between enteric CH_4_ production and feed efficiency, dry matter intake (DMI) and CH_4_ yield (ppmCH_4_/kg DMI) were assessed across different physiological groups based on key biochemical traits (Table 10). Individual DMI was calculated by subtracting feed refusals from the amount of feed offered and adjusting for the dry matter content of the total mixed ration (TMR).

Table 10 shows that cows with elevated BHB and GGT concentrations (Group 2) emitted more CH_4_ per day compared to Group 1. However, they tended to have slightly lower DMI. As a result, CH_4_ yield—representing CH_4_ emitted per kilogram of feed—was markedly higher in metabolically challenged animals.

For instance, in the BHB-based grouping, CH_4_ yield in Group 2 (23.91 ± 3.01 ppmCH_4_/kg DMI) was significantly higher than in Group 1 (11.76 ± 1.93 ppmCH_4_/kg DMI). Similar patterns were observed for AST, GGT, and LDH, with CH_4_ yields consistently higher in cows presenting altered biochemical markers (Table 10). This suggests that lower feed conversion efficiency, potentially due to metabolic stress or hepatic dysfunction, may contribute to greater CH_4_ emissions relative to intake (Table 10).

A Pearson’s correlation analysis revealed a moderate to strong negative correlation was observed between dry matter intake (DMI) and CH_4_ yield (r = −0.69), suggesting that cows consuming more feed tend to produce less CH_4_ per unit of dry matter consumed. Although the correlation did not reach conventional significance (*p* = 0.058), the trend supports previous findings. A similar trend was noted for absolute CH_4_ emission (r = −0.63), although it also did not attain statistical significance (*p* = 0.093) (Table 11).

## 4. Discussion

This study investigated the relationships between enteric methane emissions and a range of biochemical, physiological, and behavioural indicators in dairy cows during early lactation. The results revealed that metabolic status—particularly markers associated with energy balance and hepatic function—plays a key role in determining individual variation in CH_4_ output. Among the biomarkers examined, β-hydroxybutyrate (BHB) emerged as the strongest positive predictor of CH_4_ emissions, while lactate dehydrogenase (LDH) and C-reactive protein (CRP) showed inverse relationships, suggesting complex metabolic interactions between energy utilization, liver activity, and microbial fermentation.

### 4.1. Associations Between Methane Emission and Biochemical Blood Markers

Across multiple analytical approaches—including group comparisons, correlation analysis, and multiple regression—BHB consistently demonstrated a strong association with CH_4_ production. Cows with elevated BHB levels emitted 87.8% more CH_4_ compared with those within the reference range, and correlation analysis confirmed a moderate positive relationship (r = 0.54, *p* < 0.01). The regression model further identified BHB as the most significant independent predictor (β = 0.546, *p* < 0.001), while the ROC curve (AUC = 0.889) confirmed its excellent discriminatory capacity for identifying high CH_4_ emitters.

These findings align with previous studies that demonstrated that cows with ketosis exhibit alterations in the rumen microbial community, including a shift toward taxa associated with improved propionate production and reduced methanogenesis following propylene glycol treatment, which reduces BHB levels and supports energy recovery [49]. Similar relationships have been described in studies on residual feed intake (RFI), where feed-efficient (low-RFI) cows produced 9.7–15.5% less CH_4_ per day compared to inefficient (high-RFI) cows, largely due to differences in dry matter intake and metabolic efficiency [50]. During the early lactation period, cows experience an abrupt increase in energy demand after parturition, leading to intensified glucose uptake by the mammary gland and potentially triggering hypoglycaemia [51]. As a compensatory mechanism, gluconeogenesis intensifies [52], relying on substrates such as propionic acid, lactic acid, glycerol, and glucogenic amino acids [53]. When the availability of glucogenic substrates is limited, cows increasingly mobilize adipose tissue, leading to elevated non-esterified fatty acid (NEFA) and BHB levels. The oxidation of NEFA contributes to mitochondrial reactive oxygen species (ROS) production, which may further exacerbate oxidative stress and inflammation [54].

Acetate, a key short-chain fatty acid produced from fibre fermentation, has been shown to increase in cows with elevated BHB concentrations, suggesting intensified fibre breakdown or microbial shifts in the rumen under metabolic stress conditions such as subclinical ketosis [55]. Alongside this, increased formate concentrations and reduced methanol levels have been observed, suggesting alterations in fermentation pathways. Formate competes with methanogens for hydrogen and is often negatively associated with CH_4_ production, whereas methanol is a known substrate for methylotrophic methanogenesis [56]. Methanol itself may originate from either microbial fermentation or methionine metabolism and has been linked to elevated CH_4_ concentrations, particularly when ethanol is also increased, potentially due to shifts in microbial dynamics or inhibitory effects on methanol-utilizing archaea [54,57,58]. The concurrent presence of acetate and methanol—both methanogenic precursors—has been previously reported in cows with elevated BHB and NEFA, reinforcing the hypothesis that metabolic stress alters rumen fermentation in a way that favours methanogenesis [54]. Collectively, these findings support the concept that changes in systemic energy balance, particularly during negative energy balance and ketogenesis, may modulate rumen microbial populations and fermentation pathways, ultimately influencing CH_4_ output.

Ketotic cows showed a significant decrease in blood BHB levels following propylene glycol (PG) administration, which was accompanied by changes in the rumen microbiota composition, including an increase in propionate-producing genera such as *Prevotella* and *Succinivibrionaceae_UCG-001*, and a decrease in butyrate-associated taxa such as *Butyrivibrio* and *Christensenellaceae_R-7_group* [49]. These microbial shifts may reflect a redirection of fermentation pathways away from methanogenesis and toward more energetically efficient gluconeogenic routes.

The association between BHB and CH_4_ could also reflect the energetic inefficiency of metabolically stressed cows. When fat mobilization exceeds hepatic oxidation capacity, excess NEFA and ketones accumulate, increasing oxidative stress and potentially altering ruminal fermentation [59].

Moreover, the liver’s metabolic and pathological state may play a crucial role in modulating enteric CH_4_ output. Elevated BHB levels have been associated with increased ruminal butyrate production, which is subsequently converted to BHB in the liver and used systemically as an energy source. In high-methane-emitting cattle, persistently elevated BHB levels may reflect both enhanced butyrate fermentation and altered hepatic lipid metabolism, suggesting that liver function could indirectly influence CH_4_ emissions via substrate availability and energy partitioning [60].

These results are further supported by macro-level modelling studies. For instance, Mostert et al. (2020) demonstrated that subclinical ketosis (SCK) significantly contributes to increased greenhouse gas (GHG) emissions in dairy production. Their dynamic simulation model showed that each case of SCK increased GHG emissions by an average of 20.9 kg CO_2_e per ton of fat- and protein-corrected milk (FPCM), primarily due to extended calving intervals, discarded milk, reduced yield, and higher removal rates. Even cows with SCK alone (without secondary clinical diseases) produced 7.9 kg CO_2_e/t FPCM more than their healthy counterparts. These findings underscore the broader environmental relevance of early metabolic disturbances and reinforce the importance of improving metabolic resilience in transition cows to reduce both disease incidence and enteric CH_4_ emissions [61].

### 4.2. Hepatic Function Markers and Their Link to Methane Production

The current study also demonstrated moderate but consistent associations between liver enzymes and CH_4_ emissions. Cows with elevated γ-glutamyl transferase (GGT) or aspartate aminotransferase (AST) levels exhibited higher CH_4_ emissions (+25.2% and +6.4%, respectively) compared with cows within reference intervals. The strong correlations between AST, GGT, and LDH (r = 0.589 and r = 0.400, respectively) suggest that hepatic stress and impaired energy metabolism are interrelated phenomena during the transition period. In support of this, a strong positive correlation between AST and GGT (r = 0.623, *p* < 0.01) has also been reported in previous research, where these enzymes were associated with lipid mobilization and ketogenesis during early lactation [62].

As the liver plays a central role in ruminant metabolism, its sensitivity to nutritional shifts during the transition period can manifest in elevated serum enzyme activity. Increased AST and GGT concentrations are commonly observed in hepatic injury caused by metabolic overload, such as excessive lipid accumulation and lipolysis-induced lesions [63], both of which could indirectly modulate rumen function and microbial efficiency.

Interestingly, LDH showed an inverse association with CH_4_ in the regression model (β = −0.207, *p* = 0.033), indicating that cellular stress or hypoxia-related metabolic shifts might suppress methanogenesis. This could result from reduced nutrient flow to the rumen or altered redox balance in fermentative pathways. The observed inverse association between LDH and CH_4_ emissions may reflect shifts in ruminal fermentative activity under metabolic stress. LDH activity is stringently controlled by the NADH/NAD^+^ redox balance and ATP availability in ruminal microorganisms. When redox homeostasis is disrupted—such as during negative energy balance or hypoxia—lactate may accumulate transiently, potentially redirecting fermentation away from methanogenesis [64].

Studies have shown that the activities of hepatic isoenzymes LDH4 and LDH5 are significantly elevated in early lactating cows compared to those in late lactation or pregnancy, indicating increased liver stress and metabolic demand during the transition period [65]. These isoenzymes are particularly abundant in liver and skeletal muscle, and their increase may correspond with intensified lipid mobilization and compromised hepatic metabolism [66]. Additional studies have shown that cows with higher LDH activity postpartum are more likely to develop hepatic steatosis, particularly in response to high fat intake [67]. This aligns with our findings, where LDH might serve as both a marker of liver distress and a contributor to altered metabolic states affecting enteric CH_4_ emissions. Moreover, cattle fed high-concentrate diets exhibited significantly higher LDH activity than pasture-fed animals, likely due to increased metabolic load and acidotic stress [68]. Such metabolic conditions may further disrupt the rumen environment, leading to reduced methanogenesis due to lower microbial efficiency or altered hydrogen utilization. Taken together, these results suggest that elevated LDH, particularly its hepatic isoforms, may serve as a dual indicator of hepatic stress and rumen fermentative alterations. The inverse association between LDH and CH_4_ emissions observed in our study could reflect a systemic metabolic adaptation—characterized by energy redirection, oxidative imbalance, or impaired microbial function—that ultimately lowers CH_4_ production in metabolically challenged cows.

The observed three-way interaction between BHB, AST, and GGT in the mixed model (F = 6.91, *p* = 0.002) supports the hypothesis that liver metabolic status modulates CH_4_ production, particularly in the context of ketotic stress. BHB itself is derived either from amino acid degradation or from acetyl-CoA during fatty acid oxidation, and can serve as an alternative energy source for peripheral tissues [69].

### 4.3. Inflammatory and Acute-Phase Responses

Although CRP is recognized as a sensitive marker of systemic inflammation in both human and veterinary medicine [70], it did not exhibit significant differences between groups in our study and was not a significant predictor in regression or ROC models. This suggests that mild or subclinical inflammatory responses associated with metabolic strain may not be sufficient to trigger marked changes in CRP levels in dairy cows.

In the literature, CRP has shown variable sensitivity to different physiological and pathological states. Lee et al. [21] reported that CRP concentrations in dairy cows increase during peak lactation and in the presence of naturally occurring infections such as mastitis, underscoring its responsiveness to overt inflammatory processes. However, in cases of metabolic stress without acute infection, CRP may remain relatively stable, limiting its diagnostic utility.

It is important to consider that CRP is a nonspecific acute-phase protein with multiple physiological drivers, and its interpretation requires contextual evaluation alongside other metabolic, inflammatory and productivity indicators rather than in isolation [71,72]. Mild increases in CRP may occur in response to metabolic adaptation, stress, or high milk production, without necessarily reflecting clinical inflammation. Previous research has demonstrated that serum CRP concentrations correlate with milk yield and may increase physiologically during peak lactation [21], suggesting that elevated CRP can represent a lactation-associated response rather than pathology. Therefore, in the present study, CRP values were interpreted together with other metabolic biomarkers (BHB, AST, LDH), physiological data and milk productivity parameters. The absence of marked CRP elevation between groups and its weak association with CH_4_ output likely indicates that metabolic strain in early lactation did not progress to clinically relevant inflammatory activation.

Moreover, although other acute-phase proteins such as haptoglobin and serum amyloid A have been more consistently linked to metabolic imbalance in transition cows, evidence for CRP as a biomarker in such contexts remains limited [73,74,75]. This aligns with our finding that CRP showed only a weak, inverse association with CH_4_ output.

Its negative regression coefficient may reflect an indirect suppressive relationship, wherein systemic stress or inflammation subtly modulates rumen function or energy partitioning, thereby affecting CH_4_ production. Alternatively, it may reflect a compensatory dampening of inflammatory pathways in cows undergoing prolonged metabolic strain, potentially increasing their susceptibility to secondary infections or metabolic diseases [76]. This immunosuppression, in turn, may alter rumen function or nutrient partitioning, contributing to reduced fermentative capacity and lower CH_4_ production.

### 4.4. Milk Production, Composition, and Behavioural Traits

Among production and behavioural parameters, milk yield and lactose content emerged as noteworthy indicators of CH_4_ emission. ROC analysis identified milk yield (AUC = 0.823) as the strongest non-invasive predictor of high CH_4_ emission, consistent with the concept that high-producing cows typically have greater dry matter intake and ruminal fermentation, leading to elevated CH_4_ output. Indeed, a study demonstrated that increasing milk yield from 3200 to 7000 L/year per cow increased daily CH_4_ emissions from 379 to 460 g/day. Yet, CH_4_ intensity per litre of milk decreased from 35 to 21 g/L, indicating a 40% improvement in emission efficiency [77]. Similar trends were observed in the Australian dairy industry, where from 1980 to 2010, annual milk yield per cow rose by 64% (from 2889 to 5654 kg), while CH_4_ intensity dropped from 33 to 20.2 g CH_4_/kg milk [78]. These findings align with the theory that early lactation is characterized by a negative energy balance (NEB), where energy for milk production comes from body reserves rather than feed intake. As such, CH_4_ generation may be uncoupled from milk yield at this stage. In contrast, later lactation relies more heavily on dietary energy, leading to a more direct relationship between MY and CH_4_ emissions [79].

While absolute CH_4_ emissions were higher in high-yielding cows, the literature suggests that CH_4_ intensity (e.g., g CH_4_ per kg of milk) may be lower in such animals due to dilution effects. Hailemariam et al. [50] observed a negative correlation between milk production and CH_4_ intensity, indicating that improving milk yield could be a strategy to reduce emissions per unit of product. Similarly, milk lactose (AUC = 0.701) showed moderate discriminatory ability, likely reflecting rumen efficiency and carbohydrate metabolism. This is supported by genetic studies showing moderate genetic correlations (r ≈ 0.4) between milk lactose and enteric CH_4_ production, indicating that milk lactose could serve not only as a phenotypic but also as a heritable indicator of CH_4_ output in dairy cows [80]. These findings highlight the potential of incorporating lactose and other milk traits into selective breeding programs aimed at reducing CH_4_ emissions without compromising productivity.

In contrast, rumination time showed a significant negative correlation with CH_4_ (r = −0.377, *p* < 0.01), suggesting that cows with more stable rumen function and longer chewing cycles may exhibit improved feed utilization and reduced methanogenesis.

Rumination is not only a marker of digestive health but also reflects the animal’s metabolic and illness status during the transition period. Shorter rumination time has been associated with subclinical and clinical disease, making it a valuable parameter for early detection of health problems [81].

The presence of fibre in the diet increases the intensity, duration, and frequency of rumination, which in turn stimulates saliva production—an important factor for rumen pH stabilization and acetogenic fermentation [82]. Longer chewing times reduce particle size and increase microbial access, accelerating fermentation. While this supports milk fat synthesis through acetate production, it also contributes to CH_4_ generation. Still, increased rumination may indicate more effective fibre digestion and feed utilization, contributing to lower CH_4_ per unit of output [28,83,84]

Several studies have found negative associations between rumination time and CH_4_ emission. For example, cows that ruminated longer released less CH_4_ both in absolute terms and per unit of milk produced, with high-rumination cows producing 2.9% less CH_4_ per litre of milk compared to medium-rumination cows, and 4.6% less than low-rumination cows [85].

However, behavioural traits overall demonstrated weak ROC performance (AUC < 0.5), highlighting their variability and potential confounding by individual differences and environmental conditions. This aligns with conflicting evidence in the literature, as some studies did not find significant associations between rumination and CH_4_ or CO_2_ production [86].

These findings highlight complex interactions between energy metabolism, liver function, milk synthesis, and behavioural patterns. The strong enzyme correlations (AST–LDH, AST–GGT) underscore the importance of monitoring hepatic biomarkers in relation to production traits. Similarly, the methane–BHB link suggests that enteric emissions could serve as indirect indicators of metabolic stress. Negative associations between milk solids and milk temperature warrant further investigation into their physiological basis and potential implications for milk quality.

### 4.5. Integrative View and Diagnostic Implications

Overall, these findings underscore that CH_4_ emissions during early lactation indicate both digestive efficiency and overall metabolic condition. Increased levels of BHB, AST, and GGT indicate a correlation between hepatic stress, energy dysregulation, and heightened methanogenesis, whereas elevated LDH and CRP may reflect metabolic conditions that impair fermentation efficiency. The notable interaction among AST, GGT, and BHB indicates synergistic effects between hepatic function and ketone metabolism, highlighting that CH_4_ production is a multifaceted process affected by both ruminal and extra-ruminal physiology.

These findings have practical implications for CH_4_ monitoring and mitigation strategies. BHB and milk yield have proven to be effective non-invasive biomarkers for identifying cows that emit high levels of CH_4_ in field conditions. Their incorporation into precision livestock farming platforms—together with milk composition traits such as lactose and behavioural metrics like rumination—may enhance predictive models for emission intensity at the individual cow level. Conversely, hepatic enzymes and inflammatory markers exhibited diminished discriminatory power on their own yet may retain diagnostic significance when integrated into multivariate models that encapsulate the intricacies of metabolic health.

Further studies should seek to corroborate these associations in larger and more heterogeneous dairy populations, preferably incorporating individual feed intake data and direct rumen microbiota profiling. Comprehensive longitudinal tracking throughout the transition and mid-lactation phases may yield a deeper understanding of the relationship between CH_4_ emissions and metabolic adaptation and resilience. This research would enhance early warning systems for metabolic dysfunction and guide breeding or nutritional strategies to improve environmental and productive efficiency in dairy herds.

### 4.6. Limitations

This study involved clinically healthy cows and offered repeated measures during critical transition phases; however, the moderate sample size restricts the generalisability of the results. Subsequent research involving larger and more heterogeneous cohorts is essential to corroborate the identified associations and enhance diagnostic thresholds.

The ROC analyses performed in this study were exploratory. Despite the identification of several promising indicators of CH_4_ emissions, including BHB and milk yield, additional research is required to determine validated cut-off values that can be consistently utilised in precision livestock farming tools or mitigation strategies in practical field settings.

Furthermore, the panel of blood biomarkers was restricted to a fundamental set of metabolic and hepatic function indicators. The incorporation of supplementary parameters—such as insulin, glucose, NEFA, or oxidative stress markers—could yield a more refined comprehension of the physiological mechanisms affecting enteric CH_4_ production.

Likewise, physiological and behavioural indicators (e.g., temperature, rumination duration, water consumption) were examined only to a limited extent. Future studies could incorporate a wider array of activity, feeding, and stress-related metrics to elucidate the intricate interactions among metabolism, behaviour, and CH_4_ emission dynamics during early lactation.

The findings offer important insights into the complex factors influencing CH_4_ production in dairy cows; however, comprehensive longitudinal studies that incorporate metabolic, microbiological, and environmental data are essential to fully elucidate the biological foundations of individual variations in enteric CH_4_ emissions.

## 5. Conclusions

This study provides novel insights into the complex connections between metabolic status and enteric methane emissions in dairy cows during early lactation. Increased concentrations of BHB, AST, and GGT—signifying negative energy balance and hepatic stress—correlated with heightened CH_4_ emissions, implying that metabolically compromised cows may disproportionately contribute to emissions. Conversely, elevated LDH and CRP levels exhibited a negative correlation with CH_4_, potentially indicating metabolic conditions that impair fermentative efficiency.

Among the assessed parameters, milk yield and BHB were revealed as the most accurate non-invasive indicators of CH_4_ emissions under field conditions. The results underscore the potential for incorporating metabolic biomarkers, milk composition, and behavioural data into predictive models for CH_4_ monitoring. Nonetheless, additional research utilising larger sample sizes, rumen microbiota analysis, and enhanced physiological assessment is required to corroborate these associations and optimise their practical applications.

Improving metabolic health throughout the transition period may serve a dual purpose—enhancing production efficiency while reducing the environmental impact of dairy farming.

## Figures and Tables

**Table 1:** Composition of ingredients (% of dry matter) in total mixed rations for dairy cattle.

Parameters	Ingredients
Corn silage	31.0
Alfalfa grass hay	4.0
Grass silage *	10.0
Grain concentrate mash	49.0
Mineral and vitamin mix	6.0

* Grass silage contained perennial ryegrass (*Lolium perenne*), timothy (*Phleum pratense*), and meadow fescue (*Festuca pratensis*).

**Table 2:** Chemical composition of total mixed rations for dairy cattle.

Parameters	Units	Nutritional Value
Dry matter	%	50.7
Net energy lactation	Mcal/kg	1.6
Crude protein (%DM)	%	15.8
Non fibre carbohydrates (%DM)	%	38.7
Neutral detergent fibre (%DM)	%	28.3
Acid detergent fibre (%DM)	%	19.8
Ether extract (EE) (%DM)	%	4.5
Acid detergent lignin (ADL) (%DM)	%	2.3

DM—Dry matter.

**Table 3:** Reference intervals for blood biomarkers in clinically healthy dairy cows.

Biomarker	Reference Interval	Reference Interval	Source
BHB	<1.2 mmol/L	<1.2 mmol/L	[37,38,39]
CRP	<76 mg/L	<76 mg/L	[40,41]
UREA	<8.9 mmol/L	3.23–8.9 mmol/L	[12,42,43]
AST	<125 U/L	80–125 U/L	[42,44,45]
GGT	<50 U/L	25–50 U/L	[12,43,46]
LDH	<1500 U/L	1365–1795 U/L	[12,47,48]

BHB—β-hydroxybutyrate; AST—aspartate aminotransferase; GGT—gamma-glutamyl transferase; LDH—lactate dehydrogenase; CRP—C-reactive protein.

**Table 4:** Descriptive statistics of physiological, productive, and behavioural traits (mean ± standard error of mean) in cows grouped by blood BHB concentration.

Trait	Group of BHB	Mean ± SEM	ω^2^
CH_4_, ppm	1 a	286.41 ± 10.49 ^b^	−0.004
	2 b	537.89 ± 65.21 ^a^	
BHB, mmol/L	1 a	0.46 ± 0.03 ^b^	0.037
	2 b	1.52 ± 0.10 ^a^	
UREA, mmol/L	1 a	5.15 ± 0.08 ^b^	0.013
	2 b	5.60 ± 0.24 ^a^	
AST, U/L	1 a	137.46 ± 3.87	0.005
	2 b	154.71 ± 9.01	
GGT, U/L	1 a	42.53 ± 1.16	−0.003
	2 b	43.33 ± 3.57	
CRP, mg/L	1 a	9.60 ± 0.24	0.006
	2 b	9.42 ± 0.53	
LDH, U/L	1 a	1603.28 ± 35.34	0.025
	2 b	1767.44 ± 78.60	
Milk yield, kg/day	1 a	33.82 ± 0.82	−0.005
	2 b	37.29 ± 2.44	
Milk fat, %	1 a	3.95 ± 0.06	0.013
	2 b	3.73 ± 0.13	
Milk protein, %	1 a	3.58 ± 0.03	0.052
	2 b	3.47 ± 0.05	
Milk lactose, %	1 a	4.71 ± 0.01	0.019
	2 b	4.75 ± 0.03	
Milk temperature, °C	1 a	36.31 ± 0.06	0.128
	2 b	36.33 ± 0.20	
Reticulorumen temperature, °C	1 a	38.60 ± 0.11	0.008
	2 b	38.60 ± 0.32	
Activity, AU	1 a	3.37 ± 0.15	0.013
	2 b	3.38 ± 0.45	
Rumination time, min/day	1 a	490.88 ± 5.32	−0.007
	2 b	468.68 ± 14.39	
Water intake, L/hour	1 a	1.42 ± 0.40	−0.001
	2 b	1.78 ± 1.22	

Group 1 = BHB < 1.2 mmol/L (within reference interval), Group 2 = BHB ≥ 1.2 mmol/L (elevated). Different superscript letters (a, b) indicate statistically significant differences between groups (*p* < 0.05). ω^2^ indicates the effect size (random effect); BHB—β-hydroxybutyrate; CH_4_—methane; AST—aspartate aminotransferase; GGT—gamma-glutamyl transferase; LDH—lactate dehydrogenase; CRP—C-reactive protein; AU—activity units.

**Table 5:** Descriptive statistics of physiological, productive, and behavioural traits (mean ± standard error of mean) by group of AST.

Trait	Group of AST	Mean ± SEM	ω^2^
CH_4_, ppm	1 a	308.07 ± 18.19	−0.005
	2 b	329.21 ± 20.47	
BHB, mmol/L	1 a	0.58 ± 0.07	−0.006
	2 b	0.63 ± 0.06	
UREA, mmol/L	1 a	5.18 ± 0.14	−0.007
	2 b	5.23 ± 0.09	
GGT, U/L	1 a	36.24 ± 1.27 ^b^	0.121
	2 b	45.73 ± 1.41 ^a^	
CRP, mg/L	1 a	8.91 ± 0.33 ^b^	0.029
	2 b	9.89 ± 0.27 ^a^	
LDH, U/L	1 a	1410.76 ± 41.34 ^b^	0.162
	2 b	1730.91 ± 39.43 ^a^	
Milk yield, kg/day	1 a	34.04 ± 1.51	−0.008
	2 b	34.45 ± 0.93	
Milk fat, %	1 a	4.02 ± 0.10	0.005
	2 b	3.87 ± 0.06	
Milk protein, %	1 a	3.57 ± 0.04	−0.008
	2 b	3.56 ± 0.03	
Milk lactose, %	1 a	4.71 ± 0.02	−0.006
	2 b	4.72 ± 0.02	
Milk temperature, °C	1 a	36.12 ± 0.09 ^b^	0.032
	2 b	36.41 ± 0.08 ^a^	
Reticulorumen temperature, °C	1 a	38.53 ± 0.17	−0.006
	2 b	38.63 ± 0.13	
Activity, AU	1 a	3.49 ± 0.25	−0.005
	2 b	3.31 ± 0.18	
Rumination, min/day	1 a	495.12 ± 10.45	0.000
	2 b	484.13 ± 5.50	
Water intake, L/hour	1 a	1.53 ± 0.67	−0.008
	2 b	1.44 ± 0.47	

Group 1 = BHB < 1.2 mmol/L (within reference interval), Group 2 = BHB ≥ 1.2 mmol/L (elevated). Different superscript letters (a, b) indicate statistically significant differences between groups (*p* < 0.05). Omega-squared (ω^2^) reflects the effect size derived from the random-effects model; BHB—β-hydroxybutyrate; CH_4_—methane; AST—aspartate aminotransferase; GGT—gamma-glutamyl transferase; LDH—lactate dehydrogenase; CRP—C-reactive protein; AU—activity units.

**Table 6:** Descriptive statistics of physiological, productive, and behavioural traits (mean ± standard error of mean) by group of GGT.

Trait	Group of GGT	Mean ± SEM	ω^2^
CH_4_, ppm	1 a	300.70 ± 15.95 ^b^	0.034
	2 b	376.42 ± 32.87 ^a^	
BHB, mmol/L	1 a	0.59 ± 0.05	−0.001
	2 b	0.68 ± 0.09	
UREA, mmol/L	1 a	5.12 ± 0.09	0.021
	2 b	5.45 ± 0.16	
AST, U/L	1 a	129.90 ± 3.53 ^b^	0.149
	2 b	165.00 ± 7.48 ^a^	
CRP, mg/L	1 a	9.57 ± 0.26	−0.008
	2 b	9.58 ± 0.41	
LDH, U/L	1 a	1530.30 ± 31.88 ^b^	−0.167
	2 b	1867.81 ± 67.08 ^a^	
Milk yield, kg/day	1 a	34.97 ± 1.00	0.006
	2 b	32.67 ± 1.17	
Milk fat, %	1 a	3.95 ± 0.07	0.001
	2 b	3.82 ± 0.08	
Milk protein, %	1 a	3.55 ± 0.03	−0.003
	2 b	3.59 ± 0.03	
Milk lactose, %	1 a	4.71 ± 0.02	−0.004
	2 b	4.73 ± 0.02	
Milk temperature, °C	1 a	36.02 ± 0.07	−0.003
	2 b	36.24 ± 0.11	
Reticulorumen temperature, °C	1 a	38.62 ± 0.12	−0.007
	2 b	38.54 ± 0.21	
Activity, AU	1 a	3.30 ± 0.17	−0.004
	2 b	3.54 ± 0.30	
Rumination, min/day	1 a	487.45 ± 5.88	−0.008
	2 b	488.36 ± 9.82	
Water intake, L/hour	1 a	1.81 ± 0.50	0.007
	2 b	0.64 ± 0.46	

Group 1 = GGT < 50 U/L (within reference interval), Group 2 = GGT ≥ 50 U/L (elevated). Different superscript letters (a, b) indicate statistically significant differences between groups (*p* < 0.05). Omega-squared (ω^2^) represents effect size estimated from a random-effects model; BHB—β-hydroxybutyrate; CH_4_—methane; AST—aspartate aminotransferase; GGT—gamma-glutamyl transferase; LDH—lactate dehydrogenase; CRP—C-reactive protein; AU—activity units.

**Table 7:** Descriptive statistics of physiological, productive, and behavioural traits (mean ± standard error of mean) by group of LDH.

Trait	Group of LDH	Mean ± SEM	ω^2^
CH_4_, ppm	1 a	311.39 ± 20.11	−0.005
	2 b	330.54 ± 21.57	
BHB, mmol/L	1 a	0.51 ± 0.05 ^b^	0.029
	2 b	0.70 ± 0.06 ^a^	
UREA, mmol/L	1 a	5.07 ± 0.11	0.014
	2 b	5.33 ± 0.10	
AST, U/L	1 a	122.45 ± 4.83 ^b^	0.135
	2 b	153.04 ± 4.57 ^a^	
GGT, U/L	1 a	37.69 ± 1.34 ^b^	0.112
	2 b	46.36 ± 1.53 ^a^	
CRP, mg/L	1 a	9.31 ± 0.32	0.001
	2 b	9.77 ± 0.29	
Milk yield, kg/day	1 a	34.84 ± 37.20	−0.005
	2 b	33.92 ± 11.66	
Milk fat, %	1 a	4.04 ± 8.31	0.022
	2 b	3.82 ± 1.39	
Milk protein, %	1 a	3.58 ± 0.92	−0.005
	2 b	3.55 ± 0.10	
Milk lactose, %	1 a	4.71 ± 0.06	−0.006
	2 b	4.72 ± 0.04	
Milk temperature, °C	1 a	36.15 ± 0.03 ^b^	0.034
	2 b	36.44 ± 0.02 ^a^	
Reticulorumen temperature, °C	1 a	38.46 ± 0.02	0.003
	2 b	38.70 ± 0.09	
Activity, AU	1 a	3.22 ± 0.08	−0.002
	2 b	3.48 ± 0.17	
Rumination, min/day	1 a	486.73 ± 0.12	−0.008
	2 b	488.44 ± 0.22	
Water intake, L/hour	1 a	1.79 ± 0.19	−0.004
	2 b	1.24 ± 7.84	

Group 1 = LDH < 1500 U/L (within reference interval), Group 2 = LDH ≥ 1500 U/L (elevated). Different superscript letters (a, b) indicate statistically significant differences between groups (*p* < 0.05). Omega-squared (ω^2^) represents effect size estimated from a random-effects model; BHB—β-hydroxybutyrate; CH_4_—methane; AST—aspartate aminotransferase; GGT—gamma-glutamyl transferase; LDH—lactate dehydrogenase; CRP—C-reactive protein; AU—activity units.

**Table 8:** Multiple Linear Regression analysis predicting methane levels from biochemical markers.

Model		Unstandardized Coefficients		Standardized Coefficients	t	*p*	Collinearity Statistics	
		B	Std. Error	Beta			Tolerance	VIF
1	(Constant)	119.19	103.89		1.15	0.254		
	BHB	189.58	27.55	0.55	6.88	0.001	0.89	1.13
	UREA	14.94	15.63	0.08	0.96	0.341	0.87	1.15
	AST	0.44	0.41	0.10	1.07	0.286	0.60	1.68
	GGT	2.09	1.15	0.16	1.82	0.071	0.78	1.29
	CRP	1.44	5.35	0.02	0.27	0.788	0.95	1.06
	LDH	−0.10	0.04	−0.21	−2.16	0.033	0.61	1.65

B—unstandardized regression coefficient; Std. Error—standard error of the coefficient; Beta—standardized coefficient; t—t-value; *p*—probability value; Tolerance and VIF—collinearity statistics. Values were considered statistically significant when *p* < 0.05. BHB—β-hydroxybutyrate; AST—aspartate aminotransferase; GGT—gamma-glutamyl transferase; LDH—lactate dehydrogenase; CRP—C-reactive protein.

**Table 9:** Type III Tests of Fixed Effects.

Source	Numerator df	Denominator df	F	*p*
Intercept	1	40.41	6.42	**0.015**
BHB group × UREA	2	41.90	1.47	0.241
BHB group × Rumination	2	41.47	2.10	0.136
BHB group × AST	2	63.07	2.05	0.137
BHB group × GGT	2	43.22	2.28	0.114
BHB group × CRP	2	35.71	1.36	0.270
BHB group × CRP * LDH	2	41.12	1.17	0.322
BHB group × LDH	2	37.79	1.20	0.314
BHB group × UREA × Rumination	2	43.11	1.43	0.251
BHB group × AST × GGT	2	48.92	6.91	**0.002**
BHB group × AST × CRP × LDH	2	53.74	0.10	0.904
BHB group × GGT × CRP × LDH	2	46.64	0.51	0.601

df—degrees of freedom; F—F statistic; *p*—probability value. Values in bold were considered statistically significant when *p* < 0.05. BHB—β-hydroxybutyrate; AST—aspartate aminotransferase; GGT—gamma-glutamyl transferase; LDH—lactate dehydrogenase; CRP—C-reactive protein.

**Table 10:** DMI, methane emission, and methane yield across study groups in dairy cows.

Trait	Group	Mean Methane (ppm)	Mean DMI (kg/day)	Methane Yield (ppmCH_4_/kg DMI)
BHB	1	286.41 ± 10.49	24.41 ± 3.39	11.73 ± 1.69 ^a^
	2	537.89 ± 65.21	22.55 ± 2.36	23.85 ± 3.82 ^b^
AST	1	308.07 ± 18.19	23.24 ± 2.52	13.26 ± 1.48
	2	329.21 ± 20.47	22.39 ± 3.57	14.70 ± 2.52
GGT	1	300.7 ± 15.95	24.11 ± 3.08	12.47 ± 1.73
	2	376.42 ± 32.87	23.02 ± 1.65	16.35 ± 1.85
LDH	1	311.39 ± 20.11	23.86 ± 1.92	13.05 ± 1.35
	2	330.54 ± 21.57	23.43 ± 2.77	14.11 ± 1.91

^a,b^ letters indicate statistically significant differences (*p* < 0.001) in methane yield between groups. DMI—Dry matter intake; ppmCH_4_/kg DMI—Methane yield (parts per million of methane per kilogram of dry matter intake); BHB—β-hydroxybutyrate; CH_4_—methane; AST—aspartate aminotransferase; GGT—gamma-glutamyl transferase; LDH—lactate dehydrogenase.

**Table 11:** Correlations between DMI and methane emission in dairy cows.

Indices	Methane Emission (ppm)	Methane Yield (ppmCH_4_/kg DMI)
DMI	−0.63	−0.69

Correlation r (Pearson’s correlation coefficient) is significant at the *p* < 0.05 level (2-tailed). DMI—Dry matter intake; ppmCH_4_/kg DMI—Methane yield (parts per million of methane per kilogram of dry matter intake).

## Data Availability

The data provided in this study can be found in the publication.
